# Observation of tunable nonlinear effects in an analogue of superconducting composite right/left hand filter

**DOI:** 10.1038/srep14846

**Published:** 2015-10-07

**Authors:** Haiwen Liu, Jiuhuai Lei, Hao Jiang, Xuehui Guan, Laiyun Ji, Zhewang Ma

**Affiliations:** 1 Department of Information Engineering, East China Jiaotong University, Nanchang, 330013, China; 2 Tianjin Hi-Tech Superconducting Electronic Technologies Co. Ltd, Tianjin, 300384, China; 3 Graduate School of Science and Engineering, Saitama University, Saitama-shi 338-8570, Japan

## Abstract

Artificial structures with negative permittivity or permeability have attracted significant attention in the science community because they provide a pathway for obtaining exotic electromagnetic properties not found in natural materials. At the moment, the great challenge of these artificial structures in microwave frequency exhibits a relatively large loss. It is well-known that superconducting thin films have extremely low surface resistance. Hence, it is a good candidate to resolve this constraint. Besides, the reported artificial structures with negative permittivity or permeability are mainly focusing on linear regime of wave propagation. However, any future effort in creating tunable structures would require knowledge of nonlinear properties. In this work, a tunable superconducting filter with composite right/left-hand transmission property is proposed and fabricated. Its nonlinear effects on temperature and power are studied by theoretical analysis and experiments.

Metamaterials are engineered materials that consist of subwavelength electric circuits replacing atoms as the basic unit of interaction with electromagnetic radiation^1^. The history of metamaterials started in 1967 with visionary speculation on the existence of “substances with simultaneously negative values of *ε* and *μ*” by Russian physicist Viktor Veselago^2^. In 2000, following up on theoretical works^3^,^4^ done in 1990 s by John Pendry, the first metamaterial was conceived and demonstrated experimentally by Smith and colleagues at University of California, San Diego^5^. That triggered intense interest in metamaterials, in part because the ability to bend radiation in such a way has potential for creating invisibility cloaks. Since then, Smith and others^6^,^7^,^8^,^9^ have explored a host of variations on metamaterials, and a period of prosperity began. Up to now, many metamaterials have been designed and applied to invisibility cloaks^10^, perfect absorbers^11^, imaging devices^12^, terahertz devices^13^,^14^, quantum metamaterials^15^,^16^,^17^ and “illusion optics” device^18^,^19^,^20^ and so on.

Currently, loss of metamaterials is always an enormous challenge. If loss is high, the exotic electromagnetic properties such as evanescent wave amplification will be difficult to observe in practice. It is well-known that superconducting thin films with extremely low microwave surface resistance are able to produce high performance devices with low loss. So, it is a good candidate to settle the loss problem. For this reason, several superconducting metamaterial devices have been reported^21^,^22^,^23^,^24^,^25^.

Besides, the aforementioned metamaterials are mainly studied in the linear regime of wave propagation. However, any future effort in creating tunable structures, where the field intensity changes transmission properties of the composite structure, would require knowledge of nonlinear properties of such metamaterials^26^,^27^. Nonlinear properties of superconductors affect how electromagnetic waves propagate in them^28^,^29^. Research of nonlinear metamaterials is an emerging fertile arena^30^. The original approach to achieve a nonlinear response in metamaterials was realized by either engineering the elements of a metamaterial with a nonlinear component^31^ or employing a nonlinear host medium^4^. In those approaches the nonlinear response is obtained on the level of individual elements. Recently, many works on controlling metamaterial properties mechanically^32^,^33^,^34^,^35^ or thermally^36^,^37^ have been reported. In those methods, the nonlinear response mainly emerges from mutual interaction.

Here, a high-temperature superconducting (HTS) switchable filter as an analogue of right/left metamaterials is proposed and fabricated. Nonlinear effects of the HTS composite right/left hand (CRLH) filter on temperature and power are studied by theoretical analysis and experiments. In this work, we show experimentally that the off and on states of this switchable filter can be transformed by the temperature. Furthermore, an interesting exotic electromagnetic property that this artificial device at left-hand frequency is discovered experimentally to have improved power handling capability.

## Results

### The HTS CRLH filter and its circuit model

**Figure 1:**
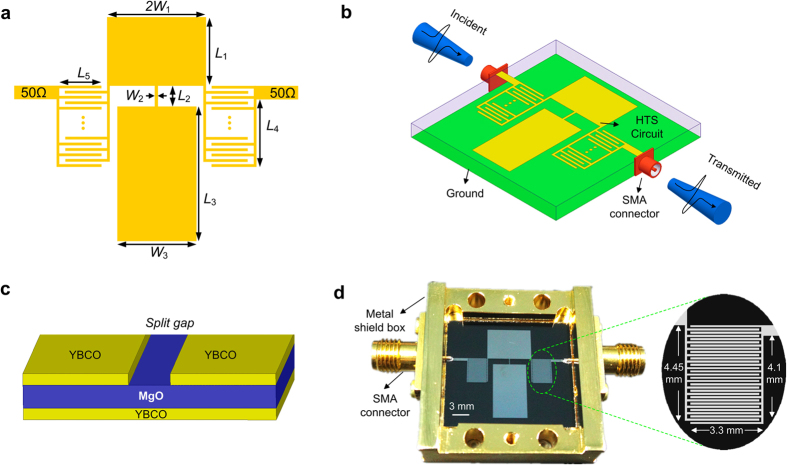
Experimental design of the HTS CRLH filter (a) Geometry and dimensions of the HTS CRLH device: *L*_1_ = 5.8, *L*_2_ = 1.1, *L*_3_ = 10, *L*_4_ = 4.1, *L*_5_ = 3.3, *W*_1_ = 3.95, *W*_2_ = 0.1, *W*_3_ = 5.9 (unit: mm). The element is patterned with 50-Ω feed lines. (**b**) Experimental configuration for transmission measurements through a fabricated device. The black curves show the measured waveforms of the incident and transmitted signal pulses. The fabricated HTS circuit is connected with SMA connectors. (**c**) Diagram of the substrate and depletion region near split gap. (**d**) Photograph of the fabricated HTS CRLH device. It is composed of SMA connector, metal shield box and HTS circuit. Its overall size is 15 mm × 16.9 mm. Scale bar has a length of 3 mm. (Substrate information: dielectric constant is 9.78, height is 0.5 mm, loss tangent is 2 × 10^−5^ at Tc = 77 K).

Geometry and dimensions of the HTS CRLH filter are shown in Fig. 1a. From this figure, the proposed CRLH filter consists of two symmetrically interdigital structures (indicated by *L*_4_ and *L*_5_) and two capacitive patches (indicated by *L*_1_, 2*W*_1_, *L*_3_ and *W*_3_) connected by a narrow microstrip line (indicated by *L*_2_ and *W*_2_). This CRLH filter is fed by a pair of 50-Ω transmission lines. The patterns indicated by yellow color are covered by superconducting thin-film materials.

In our work, HTS circuit is electrically connected using Sub Miniature version A (SMA) connectors. Its schematic diagram is depicted in Fig. 1b. This HTS circuit is fabricated on a 2-in-diameter 0.5-mm-thick MgO wafer with double-sided YBa_2_Cu_3_O_7−x_ (YBCO) films, which was sourced from THEVA Company, Germany. One diagram of substrate and depletion region near the split gap is shown in Fig. 1c. One photograph of the fabricated HTS device is given in Fig. 1d. It is composed of a HTS circuit, a pair of SMA connectors and a metal shield box.

**Figure 2:**
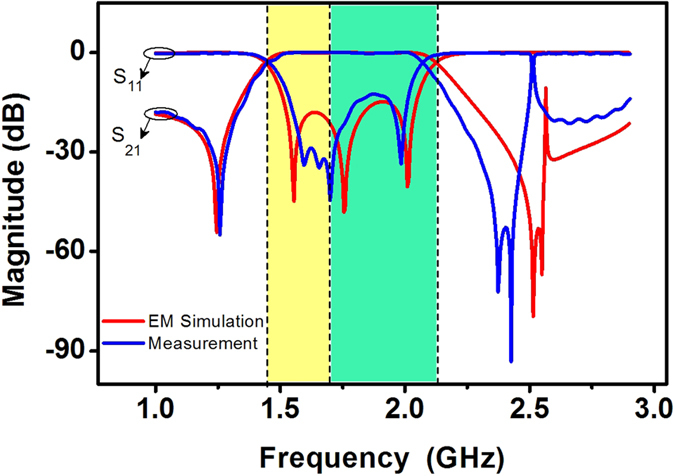
Simulation and experimental results for the fabricated HTS CRLH filter. The red curves are electromagnetic (EM) simulation results by using Sonnet software. The blue curves on left axis are the measured results at Tc = 77 K. S-parameter S_21_ is insertion loss and S_11_ is return loss. The background colors (yellow and bluish green) are in accordance with Fig. 4.

This HTS CRLH filter is designed to operate at ultra-high-frequency (UHF) band and is characterized from 1.43 to 2.13 GHz. Its −3 dB bandwidth is 40%. Its overall size is 15 mm × 16.9 mm (about 0.229 *λ*_g_ by 0.258 *λ*_g_, where *λ*_g_ is the guided wavelength at center frequency of passband). Measured results (at the critical temperature Tc = 77 K) are illustrated in Fig. 2. Measured in-band insertion loss is less than 0.22 dB and return loss is greater than 12.7 dB. It shows a good transmission performance.

**Figure 3:**
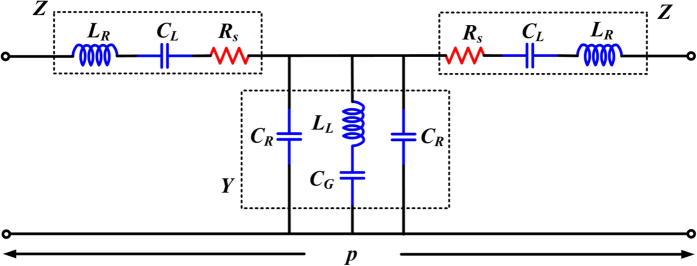
Circuit model of the proposed HTS CRLH filter. Values of the lumped-elements in the circuit model are correlated with the relevant dimensions of HTS circuit in Fig. 1a. *p* is the length of CRLH filter. *Z* is impedance and *Y* is admittance.

In order to interpret behaviors of the proposed HTS CRLH device, a circuit model is built up and shown in Fig. 3. *C*_L_ and *L*_R_ represent the coupling capacitance and parasitic inductance of interdigital structure (indicated by *L*_4_ and *L*_5_), respectively. *L*_L_ is the distributed inductances of narrow microstrip lines (indicated by *L*_2_ and *W*_2_). *C*_R_ and *C*_G_ are the distributed capacitances of wide microstrip lines (indicated by (*L*_1_, *W*_1_) and (*L*_3_, *W*_3_), respectively). *R*_s_ is the surface resistance of high-temperature superconductor thin film. *R*_s_ is set to zero when operating temperature is less than Tc. The values of *RLC* lumped-elements in this circuit model are correlated with the relevant dimensions of HTS circuit in Fig. 1a. Relative calculation formulas for extracting the circuit parameters are given in Methods.

**Figure 4:**
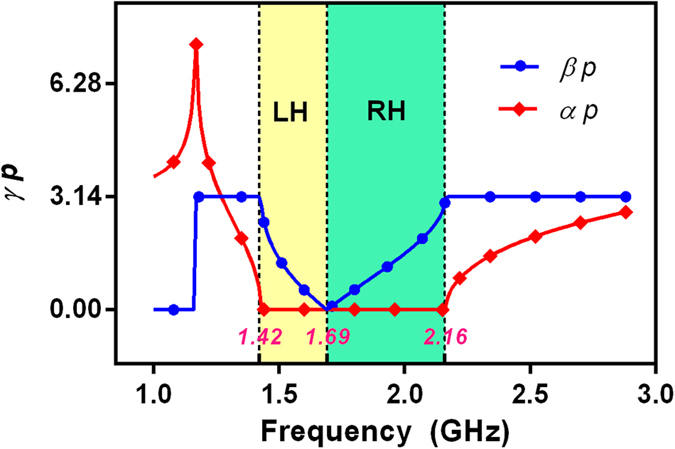
Complex propagation constant *γp*. *γ* is the complex propagation constant of the fabricated HTS CRLH filter. *p* is the total length of CRLH filter. *α* is attenuation factor and *β* is propagation constant. The red and blue curves are propagation constant (*βp*) and attenuation factor (*αp*), respectively. Circuits parameters are as follows: *L*_R_ = 4.66 nH, *L*_L_ = 1.55 nH, *C*_R_ = 5.475 pF, *C*_L_ = 1.9 pF, *C*_G_ = 12 pF, *R*_s_ = 0.

By using circuit network analysis, the complex propagation constant *γ* of this circuit model can be obtained as follows:


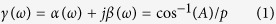


where parameter *A* is a matrix element of *ABCD*-matrix (see Methods). *p* is the total length of CRLH filter. It is a small constant. *α* is attenuation factor and *β* is propagation constant. Complex propagation constant *γ* of the fabricated HTS CRLH filter is shown in Fig. 4.

In general, the bigger attenuation factor *α*, the greater electromagnetic wave is attenuated. If attenuation factor *α* = 0, a pass-band of the proposed CRLH filter can be presented since *γ*(*ω*) = *jβ*(*ω*) is an imaginary number. Otherwise, a stop-band occurs in the frequency range when attenuation factor *α* ≠ 0. Compared with Fig. 2, it can be found in Fig. 4 that *α* ≠ 0 and a stop-band occurs within the frequency ranges of 1–1.42 GHz and 2.16–3 GHz. Nevertheless, *α* = 0 and a pass-band occurs within the frequency ranges 1.42–2.16 GHz.

On the other hand, it also can be found that the group velocity *v*_g_ < 0 (*v*_g_ = ∂*ω*/∂*β*) within the frequency range of 1.42–1.69 GHz and *v*_g_ > 0 within the frequency range of 1.69–2.16 GHz. The phase velocity *v*_p_ > 0 (*v*_p_ = *ω*/*β*) over the pass-band frequency range. In the frequency range of 1.42–1.69 GHz, *v*_g_ and *v*_p_ are antiparallel (*v*_g_*v*_p_ < 0). Generally, the group velocity *v*_g_ is associated with the direction of power flow and the phase velocity *v*_p_ is associated with the direction of phase propagation. So, in this frequency region of 1.42–1.69 GHz, the direction of power flow is opposite to phase propagation and the HTS CRLH filter shows a left-hand (LH) performance, an analogue to a left-hand metamaterial. However, in the frequency range of 1.69–2.16 GHz, *v*_g_ and *v*_p_ are parallelled (*v*_g_*v*_p_ > 0). The directions of power flow and phase propagation are the same. So, the resonator shows a right-hand (RH) performance.

**Figure 5:**
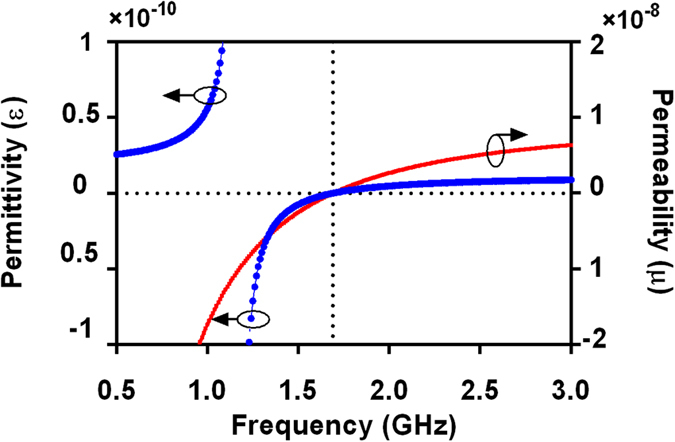
Analogical permittivity (*ε*) and permeability (*μ*) of the proposed HTS CRLH filter. Circuit parameters are as follows: *L*_R_ = 4.66 nH, *L*_L_ = 1.55 nH, *C*_R_ = 5.475 pF, *C*_L_ = 1.9 pF, *C*_G_ = 12 pF, *R*_s_ = 0.

It is well known that the properties of materials in nature can be described by defining the macroscopic parameters permittivity *ε* and permeability *μ*. This allows for media to be grouped into four categories: (1) right hand material (*ε* > 0, *μ* > 0); (2) epsilon-negative material (*ε* < 0, *μ* > 0); (3) left hand material (*ε* < 0, *μ* < 0), and (4) mu-negative medium (*ε* > 0, *μ* < 0). Figure 5 shows the analogical permittivity (*ε* = *Y/*(*jω*)) and analogical permeability (*μ* = 2*Z/*(*jω*)) with varied frequencies. From Fig. 5, it can be found that *ε* < 0 and *μ* < 0 in the frequency range of 1.42–1.69 GHz (left-hand property). In this range, the analogical Poynting vector **S** and the vector **k** are in the opposite direction^2^. *ε* > 0 and *μ* > 0 in the frequency range of 1.69–2.16 GHz (right-hand property). In this range, the analogical Poynting vector **S** and the vector **k** are in the same direction^2^.

From the analyses above, it can be concluded that this HTS filter exhibits a composite right/left-hand (CRLH) transmission performance over the passband range. In the frequency range of 1.42–1.69 GHz (left-hand property), the direction of power flow (analogical Poynting vector **S**) is opposite to phase propagation (vector **k**). And in the frequency range of 1.69–2.16 GHz (right-hand property), the directions of power flow (analogical Poynting vector **S**) and phase propagation (vector **k**) are the same.

### Nonlinear effect results on operating temperature

**Figure 6:**
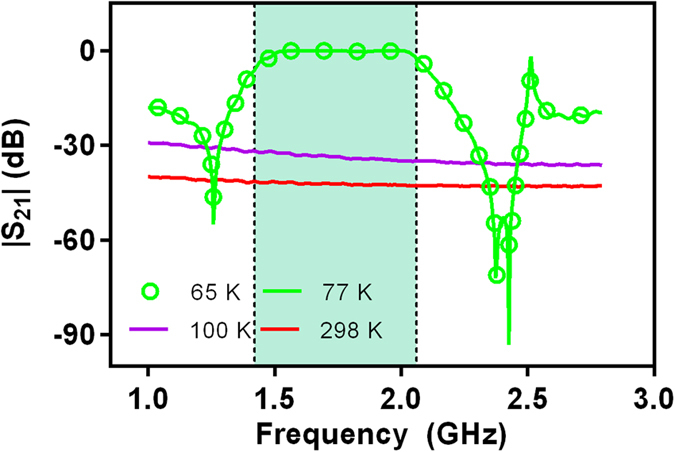
Insertion loss |S_21_| characteristics at different operating temperature. |S_21_| is the value of S-parameter S_21_ (insertion loss). Four curves shown in this figure are insertion loss characteristics at different operating temperatures (65 K, 77 K, 100 K, 298 K).

**Figure 7:**
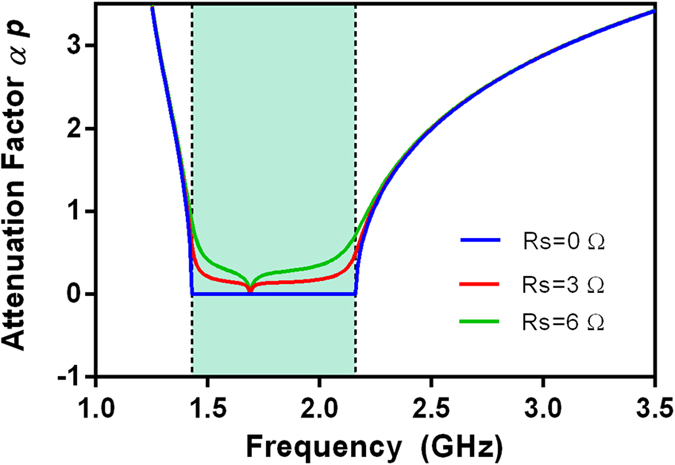
Variation of attenuation factor *αd* versus varied surface resistance (*R*_s_). *α* is attenuation factor. *p* is the length of CRLH filter. *αp* is the value of *α*  ×  *p*. Circuit parameters are as follows: *L*_R_ = 4.66 nH, *L*_L_ = 1.55 nH, *C*_R_ = 5.475 pF, *C*_L_ = 1.9 pF, *C*_G_ = 12 pF.

To further clarify microwave properties of HTS CRLH filter and make the most of superconducting properties, it is essential to understand the temperature dependence of frequency responses. Figure 6 shows the experimental frequency responses at different operating temperatures. As can be seen, the fabricated HTS CRLH filter has a steady performance when operating temperature is less than the critical temperature Tc (77 k). On the other hand, the bandpass performance from 1.42 to 2.16 GHz deteriorates when operating temperature is greater than the critical temperature Tc. This is attributed to the improved surface resistance (*R*_s_). Generally, the relation between *R*_s_ and temperature appears to be nonlinear and is shown in Methods. In superconducting technology, surface resistance *R*_s_ is extremely low when operating temperature is less than the critical temperature Tc (77 k). However, when operating temperature increases to above the critical temperature Tc, surface resistance (*R*_s_) of the HTS film is improved dramatically. The enlarged surface resistance (*R*_s_) will improve attenuation factor *α*, as shown in Fig. 7. In addition, from Fig. 6, it also can be found that the HTS CRLH filter has both bandpass performance (temperature < 77 K) and bandstop performance (temperature > 100 K). This HTS device is a good candidate for the applications of superconductor switch. Off and on states of this CRLH filter can be transformed by changing the operating temperature.

### Nonlinear effect results on input power

**Figure 8:**
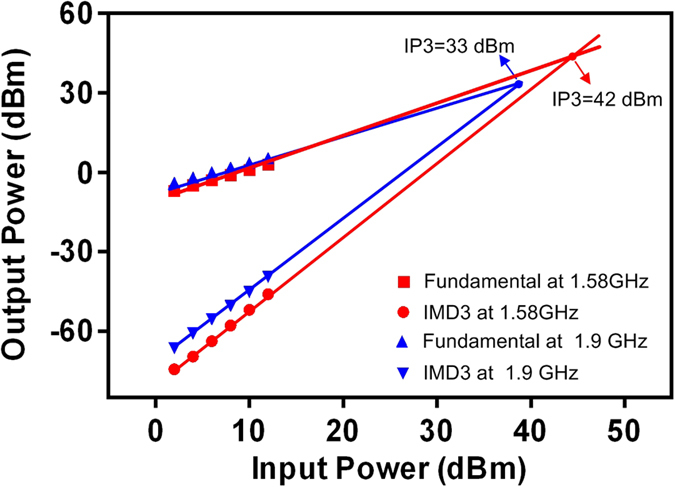
Measured IMD3 of the HTS filter for two different frequencies at 77 K. 1.58 GHz is at a left-hand frequency, and 1.9 GHz is at a right-hand frequency. IMD3 stands for the third-order intermodulation distortion. IP3 is the third-order intercept point.

For high reliable communication systems, such as digital telecommunication systems, nonlinear responses are an outstanding problem^38^. In the nonlinear regime, spurious signals are generated within passband, undermining device performance. Thus, evaluation of this characteristic is very important for HTS CRLH filter. To investigate nonlinearity of this filter, third-order intermodulation distortion (IMD3) is analyzed and measured. As a significant measurement of power handling capability, the third-order intercept point (IP3) is computed, which is defined as input power at which extrapolations of the fundamental and generated signal curves intersect. Figure 8 exhibits the input power versus output power at 77 K. Two-tone fundamental signals (1.57985 GHz and 1.58015 GHz signals for the left-hand frequency @1.58 GHz while 1.89985 GHz and 1.90015 GHz for the right-hand frequency @1.9 GHz) are input to the measured passband. IP3 of the left-hand and right-hand frequency are 42 and 33 dBm, respectively. It shows a good power handling capability. Based on the experiment results^39^, it can be found that the higher the frequency goes, the bigger the value of IP3 becomes. However in this experiment, it is interestingly found that IP3 at 1.58 GHz (left-hand frequency) is 9 dB more than that at 1.9 GHz (right-hand frequency). This means that the former can handle 8 times power as that of the latter. This experimental result reveals that the proposed HTS CRLH filter at left-hand frequency has better power handling capability. The mechanism at left-hand frequencies (*ε* < 0 and *μ* < 0) can slow electromagnetic wave, thereby increasing the interaction time with nonlinear medium embedded in it. Or they can help by concentrating the local field and thus enhancing a nonlinear response^30^.

## Discussion

In this paper, we have fabricated and characterized a CRLH filter from high-temperature superconducting YBCO films. This device has composite right/left-hand property, similar to the right/left-hand metamaterials. Its nonlinear effects on temperature and power are studied by theoretical analysis and experiments. In this work, a circuit model is built to describe and interpret the performance of this device. Surface resistance of YBCO films is taken into account to analyze the effects of temperature sensitivity. Modeling calculations are in good agreement with experimental observations and electromagnetic simulations. Also, we can find that this HTS filter is a good candidate for the applications of superconductor switch under different temperature conditions. Off and on states can be transformed by switching operating temperature. Besides, metamaterials have many exotic electromagnetic properties such as the reversal of Doppler effect^40^,^41^, the reversal of Vavilov-Cerenkov radiation^42^ and the zero index of refraction^43^, which can also be found in our structure. In this work, another exotic electromagnetic property that the left-hand frequency has better power handling capability than the right hand frequency is discovered experimentally. This finding could contribute to the research field which is in need of improving the power handling capability.

## Methods

### Fabrication and measurement processes

In this work, HTS CRLH filter was fabricated on a 2-in-diameter 0.5-mm-thick MgO wafer with double-sided YBCO films, which was sourced from THEVA, Germany. For filter patterning, a photoresist mask was prepared by photolithography, and the front-side YBCO film was etched by ion-beam milling to form the circuit structure. Filter laminate was then carefully assembled into brass housing. It was measured by an Agilent network analyzer N5230 at temperature of 77 K. Full 2-port calibration for reflection and transmission measurements is performed at room temperature.

### Circuit network analysis

Circuit model of the proposed HTS CRLH filter is shown in Fig. 3. To analyze equivalent circuit model in Fig. 3, ABCD matrix method is used. By multiplication of the unit ABCD matrices in an orderly fashion, ABCD matrix of this network is expressed as follows:


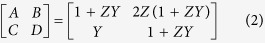


where *Z* = *R*_s_ + *jω*(*ω*^2^*L*_R_*C*_L_ − 1)/(*ω*^2^*C*_L_), *Y* = *jω*(2*ω*^2^*L*_L_*C*_G_*C*_R_ − 2*C*_R_ − *C*_G_)/(*ω*^2^*L*_L_*C*_G_ − 1).

*L*_L_ is the distributed inductances of narrow microstrip lines. *L*_L_ can be obtained by^44^:


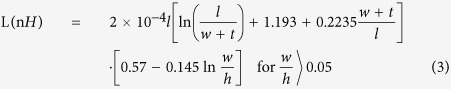


where *w*, *l* and *t* represent the length, width, and thickness of high-impedance microstrip line and *h* is the height of substrate.

*C*_R_ and *C*_G_ are the distributed capacitances of wide microstrip lines. These values can be calculated by^1^:


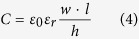


Based on the two-fluid model and BCS (Bardeen-Cooper-Schrieffer) theory, the surface resistance can be calculated by^45^:


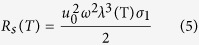


where *u*_0_ is vacuum permeability, *σ*_1_ is the real part of conductivity and *λ*(T) is magnetic penetration depth. The relation between *R*_s_ and temperature (T) appears to be nonlinear^46^.

### *N*-cell ladder network analysis

ABCD matrix of the cascade connection of *N* two-port networks, [*A*_*N*_*B*_*N*_*C*_*N*_*D*_*N*_], is equal to the product of ABCD matrices representing the individual [*A*_*k*_*B*_*k*_*C*_*k*_*D*_*k*_]. It can be described as:


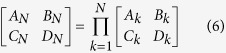


If unit cells are identical, [*A*_*k*_*B*_*k*_*C*_*k*_*D*_*k*_] = [*ABCD*]*, *
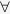
* k*, the formulas (6) can be simplified as:


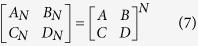


For an *N*-cell ladder network, the bigger *N is*, the more complex the electromagnetic coupling become. This strong electromagnetic coupling will change intrinsic characteristics of unit cell. To eliminate the electromagnetic coupling effects and focus on studying the characteristic of unit cell, a CRLH filter with two unit cells is proposed and fabricated.

## Additional Information

**How to cite this article**: Liu, H. *et al.* Observation of tunable nonlinear effects in an analogue of superconducting composite right/left hand filter. *Sci. Rep.* **5**, 14846; doi: 10.1038/srep14846 (2015).
